# Supplementary material for: The interactome of intact mitochondria by cross-linking mass spectrometry provides evidence for coexisting respiratory supercomplexes
Source: Mol Cell Proteomics. 2017 Dec 8;17(2):216–32. doi: 10.1074/mcp.RA117.000470 (PMC5795388; doi:10.1074/mcp.RA117.000470)
Supplement: Supplemental Data [file supp_RA117.000470_133922_0_supp_23978_fzffwf.zip › spectra_annotation/mito_spectra_annotation/230-1-4-1-4-1.pdf]

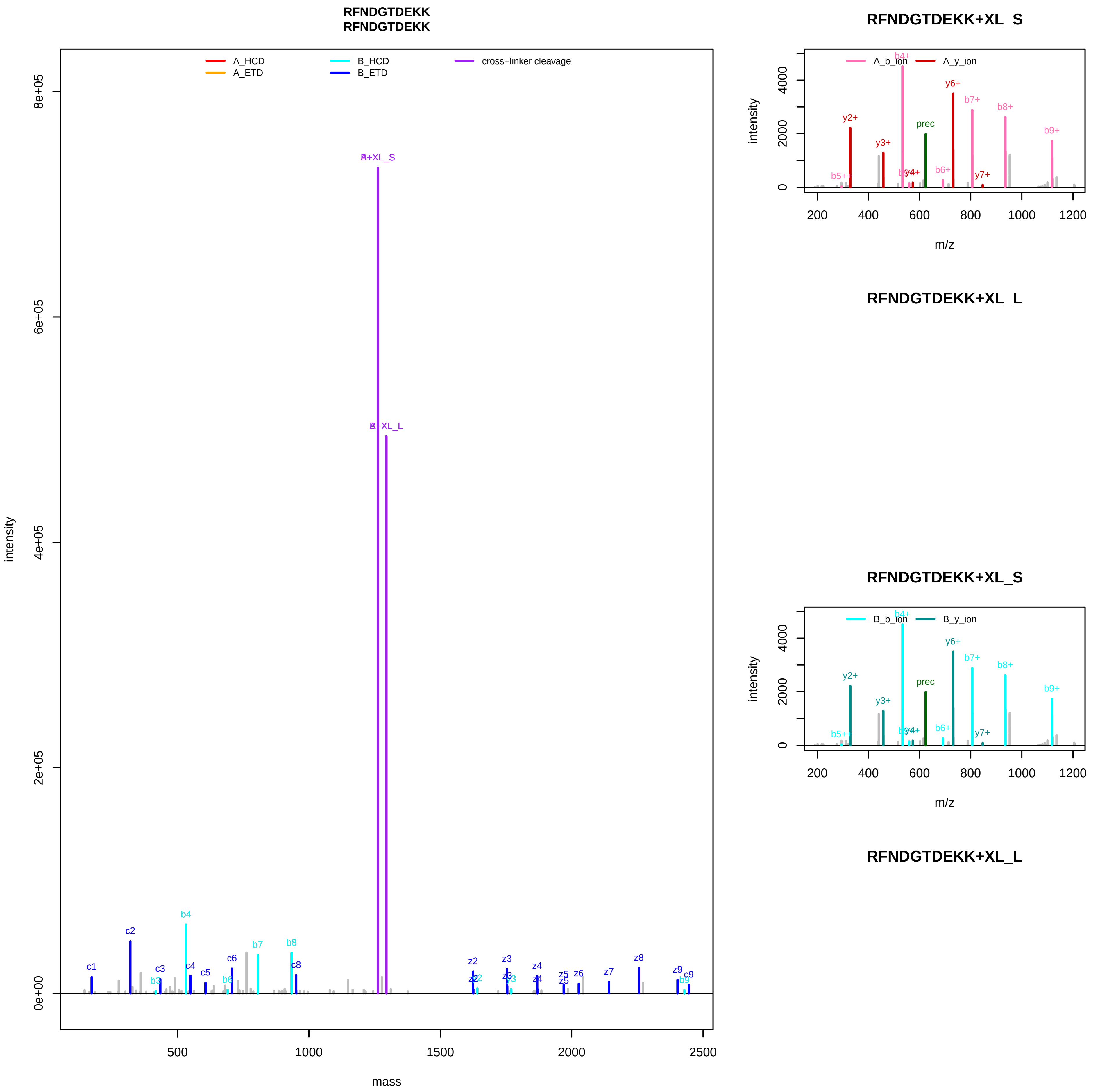
RFNDGTDEKK
RFNDGTDEKK
A_HCD
A_ETD
B_HCD
B_ETD
cross−linker cleavage
intensity
mass
8e+05
6e+05
4e+05
2e+05
0e+00
500
1000
1500
2000
2500
B+XL_S
B+XL_L
c1
c2
b3
c3
b4
c4
c5
b6
c6
b7
b8
c8
z2
z3
z4
z5
z6
z7
z8
z9
b9
c9
RFNDGTDEKK+XL_S
A_b_ion
A_y_ion
b4+
y6+
b7+
b8+
b9+
y2+
y3+
prec
b5++
y4+
b6+
y7+
intensity
m/z
0
2000
4000
200
400
600
800
1000
1200
RFNDGTDEKK+XL_L
RFNDGTDEKK+XL_S
B_b_ion
B_y_ion
b4+
y6+
b7+
b8+
b9+
y2+
y3+
prec
b5++
y4+
b6+
y7+
intensity
m/z
RFNDGTDEKK+XL_L
